# Caregiver-reported social foundations and cognitive regulation in toddlers with Down syndrome

**DOI:** 10.3389/fpsyt.2026.1803350

**Published:** 2026-07-01

**Authors:** Madison M. Walsh, Miranda E. Pinks, Kaylyn Van Deusen, Mark A. Prince, Somer Bishop, Nicole T. Baumer, Deborah J. Fidler

**Affiliations:** 1 Department of Human Development and Family Studies, Colorado State University, Fort Collins, CO, United States; 2 Psychiatry and Behavioral Sciences at the Keck School of Medicine, University of Southern California, Los Angeles, CA, United States; 3 Deptartment of Psychiatry, University of California, San Francisco, San Francisco, CA, United States; 4 Department of Pediatrics, University of Colorado Anschutz Medical Campus, Aurora, CO, United States

**Keywords:** Down syndrome, early childhood, executive function, neurodevelopmental disorders, social development

## Abstract

**Introduction:**

Down syndrome (DS) is associated with variable social developmental outcomes. Neurocognitive areas of vulnerability in DS, like executive function (EF), are a viable starting point for investigation into potential sources of individual differences in social development in children with DS.

**Methods:**

The present study investigated the association between early caregiver reported EF and social foundational skills in 107 young children with DS (*M* = 20.56 months, *SD* = 1.55). Caregivers completed the *Early Executive Functions Questionnaire (EEFQ)* and the *Communication and Symbolic Behavior Scales Developmental Profile, Infant-Toddler Checklist (CSBS)*.

**Results:**

Substantial inter-individual variability was observed among toddlers with DS along foundational social and EF dimensions. EEFQ Inhibitory control ratings were associated with all three CSBS domain scores, and Flexibility and Working Memory were associated with the Social and Symbolic domains, respectively.

**Conclusion:**

Implications for intervention and future research on regulatory vulnerabilities and social foundations in DS are discussed.

## Introduction

Down syndrome (DS) is associated with complex and variable neurobehavioral profiles during the early childhood years (1). Historically, social foundations have been described as an area of relative competence in DS (2, 3), and the development of adaptive social skills has been considered an important protective factor for many children (4, 5). A growing literature, however, has highlighted heterogeneity in social developmental outcomes in DS and identified a need to study individual differences linked to more complex profiles, such as those that involve co-occurring autism spectrum disorder (ASD; 6–8). Increased research emphasis on characterizing developmental heterogeneity in DS is motivated by the need to represent the distribution of outcomes in this population beyond characterizing group-level means (8), and an effort to increase research representation of children with DS who demonstrate more pronounced neurodevelopmental vulnerability. Moreover, identifying sources of heterogeneity in social outcomes has the potential to inform tailored and anticipatory early intervention approaches for young children with DS.

### Social foundations in Down syndrome

Although longitudinal social development trajectories in DS have not been thoroughly investigated, it is likely that differences in social outcomes can be traced back to early childhood foundations. In the general population, by the age of 18 months, children demonstrate both dyadic (3 to 6 months) and triadic (9 to 13 months) communication skills in interactions with social partners (9). Among the necessary skills for such interactions include the coordinated use of eye contact, gestures, and vocalizations for reciprocal engagement and, later, for joint attention and nonverbal requests to connect with early environments and support social learning (9). Foundations of play also emerge during this period, marked by the onset of object exploration (6 months; 10) and symbolic play (typically by 18 months; 11).

Early social communication and play foundations have been studied in neurodevelopmental conditions for several decades, with extensive research into their presentation in young children with ASD (5, 12–16). These dimensions have also been explored to a lesser extent in neurogenetic conditions linked to intellectual disability, such as DS. Existing research in DS reports nearly universal delays relative to chronological age (CA; 2, 5), but skills like joint attention (2, 5, 15, 17) and symbolic play (18) are generally reported as commensurate with overall developmental status.

It is notable, however, that the majority of social development research in DS has taken a group-level, nomothetic approach, with less emphasis on heterogeneity. Individual differences in early social communication and play have likely been masked in studies reporting group-level means and comparisons with developmentally equated counterparts. Heterogeneity may also have been underrepresented in studies wherein children with DS were specifically described as not meeting criteria for ASD (5). Thus, despite group-level characterizations of social communication and ASD features in DS and increased recognition of varied social profiles (19), little focus has been placed on how early phenotypic dimensions might be related to social outcomes in young children with this condition.

### Executive function

Neurocognitive areas of vulnerability in DS can serve as a useful starting point for exploring variability in social development in DS (20–22). In the general population, cognitive regulatory skills emerge during early childhood and organize into a set of cognitive processes known as “executive functions” (23) by the preschool years. The term executive function (EF) describes a collection of related, but dissociable cognitive processes that support goal-directed thoughts and actions. Most EF models include several key aspects: the temporary storage and manipulation of information for goal-oriented purposes (working memory), the ability to resist automatic responses in favor of more deliberate thoughts and behaviors (inhibition), and the capacity to adapt thoughts and actions based on changing context (cognitive flexibility; 24). Some models also incorporate the dimensions of emotion control or emotion regulation, which refer to the ability to modulate feelings when engaged in purposeful, goal-directed behavior (25).

EF is a known area of vulnerability for individuals with DS throughout all phases of the lifespan, even when accounting for overall degree of developmental delay (20, 26, 27). Disruptions in the development of precursors to EF are evident early in childhood in DS (2, 28) during the CA window when communication and play foundations begin to emerge, and variability in early EF likely has implications for the acquisition of social foundations. As noted above, children with DS are also at an elevated likelihood for co-occurring conditions that relate to EF, like ASD, as well as conditions known to be interrelated with EF development, like attention deficit and hyperactivity disorder (ADHD; 29). Thus, DS-related and other sources of EF disruption may have important implications for social development in DS, with potential implications for early treatment and intervention planning.

#### EF and early social communication

Although the early interplay between EF and social foundations have not been extensively investigated in DS, the relationship between these constructs has received a significant amount of consideration in neurodevelopmental disabilities research (30). Longitudinal findings link inhibition, working memory, and cognitive flexibility to joint attention skills in children with ASD aged 18 to 36 months (31). Mechanistically, early aspects of inhibition are hypothesized to facilitate social orienting and subsequent joint attention through a child’s ability to select and attend to some stimuli, like social partners, over other exogenous stimuli (30). Early flexibility in attention also facilitates the visual engagement and disengagement necessary to follow another person’s gaze, or to alternate visual attention between an object/event of interest and a social partner (30). There is mounting evidence supporting these hypothesized associations, with converging reports of longitudinal associations between early infant visual attention and later social communication and joint attention skills across study samples and clinical populations (12, 32, 33), but this association has not yet been investigated in DS.

#### EF and early play skills

In addition to its role in facilitating social communication, EF has also been hypothesized as foundational for the development of play (34). Foundations of working memory and inhibition help infants organize exploratory behavior with objects and facilitate the development of representations of their properties and affordances (35, 36). Later, EF contributes to more advanced play skills involving pretense, or the ability to let one thing symbolize another (37, 38). Children without disabilities typically engage in symbolic pretense by around 18 months of age, such as attributing characteristics of living things to inanimate objects. They then progress to object substitutions (e.g., a block used as a phone) and more complex imaginary scenarios by preschool age. EF is thought to support this development by inhibiting incoming information about the real world in favor of imagined scenarios and by increasing flexibility to manage conflicting representations of reality and pretense (37, 38).

#### EF and social foundations in DS

Despite limited systematic research into the link between EF and social foundations in DS, existing findings suggest a potentially important connection between these areas in this population. A previous study of a small sample of toddlers with DS found a link between triadic communication and performance on an object retrieval task that involved executive-related planning and organizing skills (2). A more recent study showed that parent ratings of infant regulatory function were predictive of communication skills six months later in DS (28). To date, however, there has been no investigation into the association between foundational EF and social presentations in toddlers with DS. Identifying such an association has the potential to inform anticipatory approaches to social interventions in DS by targeting both foundations of goal-directed behavior as well as early social behaviors. New discovery regarding EF and social development in DS may also inform the study of early social vulnerabilities in children with cognitive delays more broadly.

The current study investigated the potential interplay between early EF, social communication, and symbolic play skills in 1-year-old children with DS. Caregivers completed questionnaires assessing foundational aspects of child communication, play, and early EF. Key domains were characterized and their interrelationships were examined, with potential implications for early intervention in DS.

## Methods

### Participants

Participants were 107 young children with DS between 17 and 24 months of age (*M* = 20.59, *SD* = 1.57, female = 52.3%) and their caregivers. Mental age (MA) equivalents were estimated using scores from the Bayley-4 Cognitive Domain (39) (*M* = 11.58, *SD* = 2.71). To be eligible to participate, children were required to have a confirmed medical diagnosis of DS and caregivers were required to speak and understand English. Children were excluded from the study if they had complete hearing and/or vision loss; however, participants were not excluded based on other co-occurring medical or other neurodevelopmental conditions. No participants reported co-occurring ASD or ADHD at the time of data collection, likely due to their young chronological ages. Participant demographics are reported in **Table 1**.

**Table 1:** Participant demographics.

Child characteristics	n	%
Sex		
Male	51	48%
Female	56	52%
Race		
White	75	70%
Multiple	14	13%
Black or African American	8	7%
Asian	2	2%
Other	2	2%
American Indian or Alaska Native	1	1%
Native Hawaiian or Other Pacific Islander	1	1%
Did not report	4	4%
Ethnicity		
Not Hispanic or Latino	76	71%
Hispanic or Latino	28	26%
Did not report	3	3%
Down syndrome Type		
Complete Trisomy 21 (Nondisjunction)	98	92%
Mosaic Trisomy 21	1	1%
Translocation Trisomy 21	2	2%
Not Sure	6	5%
Caregiver characteristics	n	%
Education (highest level attained)		
Primary school	3	3%
Secondary school	17	16%
Associate/university/post-graduate degree	87	81%
Household income		
Less than $50,000	13	12%
$50,000 - $100,000	28	26%
More than $100,000	58	54%
Did not report	8	8%

### Procedure

Data were collected as part of a multi-site longitudinal study examining early development in DS. Informed consent was obtained before study procedures began. All study procedures were approved by the Western Copernicus Group Institutional Review Board. Participants were recruited from across the continental United States and Canada through flyers distributed by regional Down syndrome organizations, online advertisements, Down syndrome clinics, and word of mouth. Research visits were conducted in locations convenient for families in order to remove barriers to participation. Therefore, research visits were conducted in clinical, laboratory, and community settings, including participants’ homes. During each research visit, children completed a battery of assessments with trained research associates. Caregivers completed digital questionnaires regarding their child’s development during the visit or within two weeks of the in-person visit.

### Measures

#### The Bayley Scales of Infant and Toddler Development, Fourth Edition (Bayley-4)

The Bayley-4 is a direct assessment of five areas of development (cognitive, receptive language, expressive language, fine motor, and gross motor) in children aged 16 days to 42 months (39). The Bayley-4 has been normed on census data in the United States and has been used in clinical populations, including children with intellectual and developmental disability (39). The measure has high internal reliability (.93 to .95) and test-retest reliability (.81 to .84). Age equivalents to estimate overall cognitive status and both raw and standard scores were included in analyses, as appropriate. Scores from the Bayley-4 Cognitive Domain were missing for one participant due to participant fatigue.

#### The Early Executive Functions Questionnaire (EEFQ)

The EEFQ is a 31-item caregiver-report questionnaire that assesses EF foundations in infants and toddlers aged 9 to 30 months (40) that demonstrates convergent validity with other EF and temperament measures in young children with DS (Walsh et al., under review). Caregivers report on occurrences of their child’s behaviors in the previous two weeks. The measure uses Likert-type items with seven response options (“Never,” “Very rarely,” “Less than half the time,” “About half the time,” “More than half the time,” “Almost always,” and “Always”). Response choices also include “Does Not Apply,” which is selected when a caregiver has not had the opportunity to observe their child in that situation in the last two weeks. This response option is differentiated from “Never,” because a “Never” response indicates that the opportunity to observe a behavior was present and the child did not use that behavior (e.g., “quiet down when you ‘shushed’ them so as not to disturb others [such as in a library or church, or on a bus]”). Higher EEFQ scores indicate more advanced EF development.

EEFQ subscale scores are calculated using the mean of the item scores comprising each subscale, excluding “Does Not Apply” items and skipped or missing items; item scores are reversed when designated as such. The EEFQ subscales were derived through exploratory factor analysis and confirmatory factor analysis (40). The EEFQ authors report that the subscales show limited floor and ceiling effects, good internal consistency, and convergent validity with caregiver-report measures of attentional control (40). In the current sample, items on the Inhibitory Control and Regulation subscales demonstrated acceptable internal reliability with Cronbach’s alpha at .70 and .84, respectively. Scores on the Flexibility and Working Memory subscales demonstrated lower reliability with Cronbach’s alphas at .49 and .65, respectively. For item-level missingness, EEFQ scores were calculated as average item scores based on the total number of items completed; as a result, all participants had EEFQ domain scores.

#### The Communication and Symbolic Behavior Scales Developmental Profile: Infant Toddler Checklist (CSBS)

The CSBS is a caregiver-report measure designed to identify delays in social communication, expressive language, and symbolic functioning in children with a functional communication age between 6 and 24 months (41). Caregivers respond to 24 items with answer choices of “Not Yet,” “Sometimes,” and “Often.” Seven cluster scores (Emotion and Eye Gaze, Communication, Gestures, Sounds, Words, Understanding, and Object Use) are calculated from item responses. In addition to a total score, three composite scores are calculated from the clusters: Social (composed of the Emotion and Eye Gaze, Communication, and Gestures clusters), Speech (composed of the Sounds and Words clusters), and Symbolic (composed of the Understanding and Object Use clusters). The CSBS was normed on a sample of children without neurodevelopmental conditions and has age-based norms for generating percentile scores up to 24 months. It has been validated for use for individuals with neurogenetic conditions (42, 43) and has been used in previous studies to assess social communication in infants and toddlers with DS (28).

Cronbach’s alpha was calculated in the current sample to evaluate the internal consistency for each composite score and was found to be acceptable for the Social composite (.81) and the Symbolic composite (.70). Internal consistency was lower for the Speech composite (.58). In the case of item-level missingness, missing scores were recoded as zero (“Not Yet”), as non-response was interpreted as a skill not observed. Item-level missingness was not common on the CSBS; five participants did not complete one item, and three participants did not complete two items. Both raw scores and percentile scores were included in analyses, with higher scores for both connoting more advanced skill acquisition in a given area. Percentile scores were used for descriptive purposes, and raw scores were used in subsequent analyses.

### Analysis plan

Descriptive statistics were generated to interpret the distribution of presentations for the CSBS and EEFQ. CSBS percentile scores were calculated for descriptive analyses. CSBS and EEFQ raw item scores were examined for skew. Associations among Bayley-4 scaled scores, CSBS raw composite scores, and EEFQ subscale scores were examined using pairwise Pearson correlations. Pairwise Pearson correlations were computed between CSBS raw cluster and composite scores and EEFQ subscale scores, and a subset matrix containing CSBS x EEFQ associations was visualized in a correlogram using the ‘corrplot’ package in R to illustrate the strength and direction of associations (44). To account for multiple comparisons across the correlation matrix, *p*-values were adjusted using the Benjamini–Hochberg false discovery rate (FDR) procedure. Significant associations (adjusted *p* <.05) were labeled in the correlogram with asterisks denoting significance level. CSBS Social, Speech, and Symbolic raw composite scores were each regressed onto the four EEFQ domain scores in separate linear regression models, with Bayley-4 Cognitive raw scores included as a covariate to account for developmental variance in CSBS outcomes. Multicollinearity among EEFQ predictors was evaluated using variance inflation factors (VIFs). VIF values greater than 5 were considered indicative of potential multicollinearity concerns.

## Results

### Descriptive statistics

#### CSBS scores

For the CSBS Social composite, scores ranged from the 1^st^ to 84^th^ percentile (median = 9^th^ percentile, *M* = 16.5, *SD* = 21.11). Speech composite scores ranged from the 1^st^ to 50^th^ percentile (median = 9^th^ percentile, *M* = 9.46, *SD* = 9.86). Symbolic Composite scores ranged from the 1^st^ to 63^rd^ percentile (median = 5^th^ percentile, *M* = 10.67, *SD* = 13.79). Total CSBS raw scores ranged from 14 to 52 (*M* = 32.42, *SD* = 8.22). Raw scores were utilized in all subsequent analyses.

##### Item level patterns

Although several individual items demonstrated positive or negative skew, variability was retained across the broader measure as demonstrated by the composite score descriptive statistics. Of the 24 items in the CSBS, five items (20.8%) had a skew less than –1, indicating skew toward skill acquisition in this sample, including: “Do you know when your child is happy and when your child is upset?”, “Does your child smile or laugh while looking at you?”, “When you are not paying attention to your child, does he/she try to get your attention?”, “When you call your child’s name, does he/she respond by looking or turning toward you?”, and “Does your child show interest in playing with a variety of objects?”. Two of the 24 CSBS items (8.3%) had a skew greater than 1, indicating skew toward lack of skill acquisition in the sample, including: “Does your child nod his/her head to indicate yes?” and “Does your child put two words together (for example, *more cookie*, *bye-bye Daddy*)?”.

The remaining 17 CSBS items demonstrated heterogeneous patterns across the sample. Items with at least 10% of caregivers reporting each of the response options (indicating variability across the sample) included: “Does your child let you know that he/she needs help or wants an object out of reach?”, “Does your child do things just to get you to laugh?”, “Does your child try to get you to notice interesting objects - just to get you to look at the objects, not to get you to do anything with them?”, “Does your child pick up objects and give them to you?”, “Does your child show objects to you without giving you the object?”, “Does your child point to objects?”, “Does your child string sounds together, such as *uh oh*, *mama*, *gaga*, *bye bye*, *bada*?”, “About how many blocks (or rings) does your child stack?”, and “Does your child pretend to play with toys (for example, feed a stuffed animal, put a doll to sleep, put an animal figure in a vehicle)?” Complete item-level descriptive statistics are provided in **Supplementary Table 1**, including item means, standard deviations, and skew values.

##### Bayley-4 associations

CSBS raw composite scores generally demonstrated small to moderate positive associations with Bayley scaled scores (see **Table 2**). Associations with the Social composite ranged from .28 (Bayley-4 Gross Motor) to .53 (Bayley-4 Expressive Communication). Associations with the Speech domain ranged from.08 (Gross Motor) to .39 (Expressive Communication). Associations with the Symbolic composite ranged from .31 (Gross Motor) to .50 (Fine Motor).

**Table 2:** Correlations for Bayley Scaled Scores, CSBS Raw Scores, and EEFQ Subscale Scores.

Variable	1	2	3	4	5	6	7	8	9	10	11	12
1. Bayley Cognitive SS												
2. Bayley Expressive SS	0.44***											
3. Bayley Receptive SS	0.41***	0.45***										
4. Bayley Fine Motor SS	0.63***	0.57***	0.35**									
5. Bayley Gross Motor SS	0.48***	0.23*	0.29**	0.47***								
6. CSBS Social	0.39***	0.53***	0.35**	0.50***	0.28**							
7. CSBS Speech	0.28**	0.39***	0.19	0.26*	0.08	0.52***						
8. CSBS Symbolic	0.43***	0.42***	0.35***	0.50***	0.31**	0.62***	0.43***					
9. EEFQ IC	0.43***	0.33**	0.21*	0.32**	0.27**	0.47***	0.40***	0.43***				
10. EEFQ Flex	0.32**	0.28**	0.22*	0.38***	0.29**	0.50***	0.37***	0.33**	0.66***			
11. EEFQ WM	0.25*	0.25*	0.21*	0.19	0.27**	0.31**	0.20*	0.42***	0.46***	0.53***		
12. EEFQ Reg	0.09	-0.07	0.04	0.02	-0.02	0.02	0.02	-0.03	0.15	0.17	0.06	
13. EEFQ Total	0.40***	0.29**	0.25*	0.33**	0.29**	0.46***	0.35***	0.41***	0.82***	0.83***	0.72***	0.47***

SS, Scaled Score; IC, Inhibitory Control; Flex, Flexibility; WM, Working Memory; Reg, Regulation. Statistical significance is based on FDR-adjusted *p*-values. Significance levels indicated as *p < .05, **p < .01, ***p < .001.

#### EEFQ scores

When comparing across EEFQ domain average item scores, relatively higher means (indicating more advanced EF) were observed for the Working Memory (*M* = 4.72, *SD* = .86) and Regulation subscales (*M* = 5.74, *SD* = .83), and relatively lower means were observed for the Inhibitory Control (*M* = 3.97, *SD* = .94) and Flexibility (*M* = 3.73, *SD* = .83) subscales. The items with highest average scores in the sample, connoting greater competence in these areas, were “return to being calm/happy within 3 minutes of a small frustration”, “go after something they wanted (e.g. your phone or the remote control for the TV) even after you had just hidden it from view”, and several reverse scored items: “get upset during a fun activity and need to be soothed,” “show anger (e.g., screaming, shouting, or lashing out) after they couldn’t have something they wanted, and stay angry for more than a minute”, and “show anger (e.g., screaming, shouting, or refusing to move) after being taken away from somewhere fun, and stay angry for more than a minute”.

The items with lowest scores on average, connoting less competence in these areas, were: “grab or point to the odd one out in a group (e.g., a bright piece of clothing in a pile of washing, or a ball among toy animals)”, “use everyday objects to solve problems without being shown (e.g., if something was out of reach dragged over a box to climb on, or got a stick to poke it)”, “stop reaching completely for something when you said “no/don’t touch” or similar”, “try a different way to complete a tricky task without being shown (e.g., when putting shapes in a shape sorter tried different holes)”, and one reverse-scored item: “approach or reach for something that they have been repeatedly told not to touch (such as electrical sockets or the oven)”. Item-level descriptive statistics are provided in **Supplementary Table 2**, including item means, standard deviations, and skew values.

##### Bayley-4 associations

EEFQ subscale scores were generally positively associated with Bayley-4 scaled scores (see **Table 2**). The strongest magnitude association with Inhibitory Control scores was .43 (Bayley Cognitive), with other scores ranging from .21 to .33. Associations with Flexibility ranged from .22 (Receptive Communication) to .38 (Fine Motor). Associations with Working Memory ranged from .19 (Fine Motor) to .27 (Gross Motor). Associations with Regulation ranged from -.07 (Expressive Communication) to .09 (Cognitive).

### Associations Between CSBS and EEFQ ratings

#### Correlation matrix visualization

**Figure 1:**
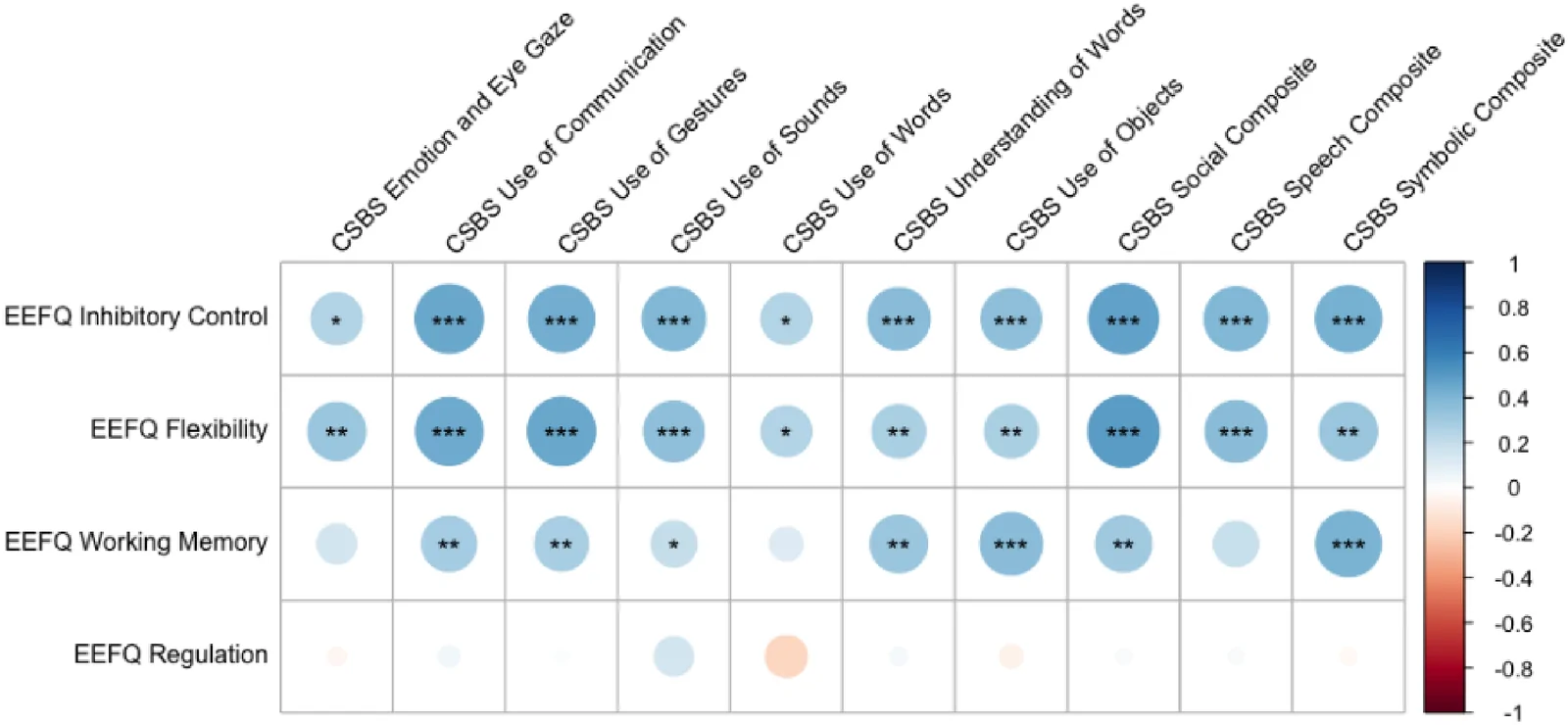
Correlogram of partial correlations between EEFQ average item scores and CSBS raw scores. Statistical significance is based on FDR-adjusted *p*-values. Significance levels indicated as *p < .05, **p < .01, ***p < .001.

The correlogram in **Figure 1** illustrates the partial correlations among the four EEFQ subscale scores and the seven CSBS raw cluster scores and three CSBS raw composite scores. Strength of association is indicated by the size of the circles; positive correlations are represented in shades of blue and negative correlations are represented in shades of red, with darker hues reflecting stronger associations. FDR-adjusted significant correlations are labeled in the correlogram (**Figure 1**). Significant associations were observed between the EEFQ Inhibitory Control and Flexibility subscales with all three CSBS composite scores (Social, Speech, and Symbolic; *r*’s between .33-.5). Additional associations were observed between EEFQ Working Memory and various CSBS domains (see **Figure 1**). Notably, negligible associations were observed between the EEFQ Regulation scale and all CSBS scores. Correlation coefficients with confidence intervals (CI) are provided in **Supplementary Table 3**.

#### Regression analyses

To better model these associations, CSBS Social, Speech, and Symbolic raw composite scores were regressed on to the EEFQ domain scores in three separate regression models, each controlling for Bayley-4 Cognitive raw scores. Examination of VIFs indicated that multicollinearity was not a concern, with all VIF values below 2 (range = 1.04 - 1.97). The overall model for CSBS Social composite scores was significant, *F* (5, 91) = 10.14, *p* <.001, and the predictors explained 35.8% of the total variance (*R^2^* = 0.36). Flexibility and Bayley-4 Cognitive scores were significant positive predictors of the CSBS Social composite (*B* = 1.51, *p* = .02 and *B* = 0.11, *p* = .004, respectively). The model predicting the CSBS Speech composite scores was also significant, *F*(5, 91) = 5.75, *p* <.001, with EEFQ domains and Bayley-4 Cognitive scores accounted for 24.0% of the variance (*R^2^* = 0.24). EEFQ Inhibitory Control was a significant positive predictor of CSBS Speech (*B* = 0.71, *p* = .02). The model predicting CSBS Symbolic composite scores was statistically significant as well, *F*(5, 91) = 11.81, *p* <.001, and accounted for 39.4% of the variance in Symbolic scores (*R^2^* = 0.39), with Inhibitory Control (*B* = 0.77, *p* = .04), Working Memory (*B* = 0.99, *p* = .007), and Bayley Cognitive scores (*B* = 0.10, *p* <.001) predicting CSBS Symbolic composite scores. Regression results are reported in **Table 3**.

**Table 3:** Multiple regression predicting CSBS outcomes from EEFQ domains.

Predictor	*B*	*SE*	*t*	*p*	95% CI
CSBS Social					
Intercept	2.75	3.70	0.75	.458	[-4.59, 10.10]
EEFQ IC	0.77	0.55	1.41	.162	[-0.32, 1.85]
EEFQ Flex	1.51	0.63	2.40	.018*	[0.26, 2.77]
EEFQ WM	0.21	0.53	0.39	.695	[-0.84, 1.25]
EEFQ Reg	-0.44	0.46	-0.95	.342	[-1.36, 0.48]
Bayley Cog	0.11	0.04	2.95	.004**	[0.04, 0.18]
CSBS Speech					
Intercept	2.66	2.00	1.33	.188	[-1.32, 6.64]
EEFQ IC	0.71	0.30	2.39	.019*	[0.12, 1.29]
EEFQ Flex	0.56	0.34	1.63	.106	[-0.12, 1.24]
EEFQ WM	-0.18	0.29	-0.64	.526	[-0.75, 0.39]
EEFQ Reg	-0.30	0.25	-1.20	.235	[-0.80, 0.20]
Bayley Cog	0.02	0.02	1.10	.275	[-0.02, 0.06]
CSBS Symbolic					
Intercept	-1.51	2.51	-0.60	.549	[-6.49, 3.47]
EEFQ IC	0.77	0.37	2.08	.041*	[0.03, 1.50]
EEFQ Flex	-0.30	0.43	-0.69	.492	[-1.15, 0.56]
EEFQ WM	0.99	0.36	2.78	.007**	[0.28, 1.70]
EEFQ Reg	-0.42	0.31	-1.34	.184	[-1.04, 0.20]
Bayley Cog	0.11	0.02	4.22	<.001***	[0.06, 0.15]

*B*, unstandardized coefficient; *SE*, standard error; CI, confidence interval; IC, Inhibitory Control; Flex, Flexibility; WM, Working Memory; Reg, Regulation; Cog, Cognitive. * *p* < .05, ** *p* < .01, *** *p* < .001.

## Discussion

This study examined the association between EF foundations and early social dimensions in a sample of 1-year-old children with DS. Caregivers of toddlers with DS provided ratings of early EF, social communication, and aspects of play development. Substantial heterogeneity was observed within the sample along each dimension. Caregiver ratings of inhibitory control, flexibility, and working memory, but not emotion regulation, were associated with various aspects of social communication and symbolic thinking in this sample. These cross-sectional findings can serve as a step toward understanding individual differences in social competencies in this population, with potential implications for subsequent social outcomes.

The present findings replicate patterns reported in other clinical populations. In children with ASD, EF ratings are associated with communication (45), social behavior in real-world settings (46), and social skills (47). Although reports vary, it is estimated that approximately 18% of individuals with DS also meet criteria for ASD (48), and yet the emergence of DS+ASD remains poorly understood. Advancing our understanding of individual differences in early social foundations could also have important implications for the early identification of ASD in children with DS. Because EF is a known area of vulnerability for many individuals with DS, evaluation of these associations in this population has the potential to explain heterogeneity in outcomes. In the present study, the association between ratings of inhibition and social foundations was evident at this early stage of development in DS. Notably, EEFQ Inhibition ratings were significantly associated with all three CSBS composite scores: the Social, Speech, and Symbolic dimensions. Additional significant predictors differed across the three CSBS domain models, with Working Memory significantly associated with the Symbolic composite, and Flexibility significantly associated with the Social composite.

The results presented in this paper expand on previous DS research that shows short-term longitudinal links between caregiver ratings of early regulatory function and later ratings of social communication during the transition to toddlerhood (28). Although the present study examines concurrent associations, these findings replicate an early connection between regulatory processes and social development in young children with DS when assessed through proxy reports. Additionally, this study leverages the availability of a new assessment tool, the EEFQ, designed to measure early foundations of EF and its development across infancy. The convergence between this work and previous studies involving other cohorts of infants with DS, as well as broader early measures of regulation, suggests that these associations are consistent across different measures and developmental periods during early childhood in DS.

### Hypothesized mechanisms

The current findings raise further questions about the proposed links between EF and social skills early in development. It has long been hypothesized that inhibition is crucial for developing joint attention and acts as a foundation for alternating focus between a social partner and an object or event (30). The observed cross-domain relationships between Inhibition ratings and CSBS Speech and Symbolic composite dimensions support and potentially expand upon this previously suggested mechanism discussed in the ASD literature. Other findings reported in the general literature were also replicated, including the link between Working Memory ratings and Symbolic composite scores (49), which is plausible since representing and using symbols depends on actively manipulating and transforming incoming information from the environment. Likewise, the connection between Flexibility and Social domain scores is logical in that early social interactions involve spontaneous exchanges with social partners that are often unpredictable and require the ability to adapt and respond to changes in social dynamics.

The present findings also raise broader questions regarding the ongoing interaction between EF and the acquisition of social skills throughout childhood in DS. Early challenges with EF foundations may have cascading effects onto social learning and social adaptation to varying degrees among children with DS, especially as the cognitive and self-regulatory demands associated with relationship formation increase during the middle childhood and adolescent years. Accordingly, delays in these cognitive foundations are likely to contribute to individual differences in social presentations in this population throughout childhood.

#### Direction of effects

The present findings align with previous longitudinal data and theoretical models that place early cognitive and attention regulation as foundational for the acquisition of later social communication. Early inhibition, for example, has been identified as a key precursor to the ability to suppress attention toward exogenous sources of information in favor of more informative social sources. In this way, inhibition and early attention control are thought to facilitate the acquisition of dyadic and triadic social interaction skills. However, as development continues to unfold, the opposite direction of effects is plausible as well. Formulating social attachments to caregivers, for instance, is likely to facilitate aspects of emotion and behavior regulation, thereby facilitating subsequent goal directedness and organized purposeful behavior (50). Social attachments and early triadic interactions may be an important facilitator of the acquisition of later EF skills. Indeed, recent models of parent-child interactions emphasize the importance of characterizing ‘co-regulatory’ dynamics, wherein certain parent-child dyad patterns impact children’s self-regulatory processes (51). Future work may benefit from a more dynamic understanding about how these two dimensions exert ongoing effects in bidirectional and more granular ways via longitudinal and direct assessment approaches.

Another critical dimension to discern relates to the elevated likelihood of co-occurring ADHD in children with DS (29). Much like growing body of literature on ASD in DS, a similar set of investigations has identified a subgroup of children with DS who meet criteria for co-occurring ADHD. Given the role of executive dysfunction in ADHD diagnoses in the general population, a critical next step in this line of research relates to exploring the interrelatedness of ADHD and ASD symptomatology, with a focus on direction of effects. This line of research is especially timely in the context of increased recognition of symptom overlap across the two conditions during early childhood in the general population (52).

### Implications

Studying the early origins of within-DS heterogeneity in social presentations has the potential for both basic scientific and translational relevance. From a basic science perspective, DS offers a unique opportunity to prospectively study early social development in the context of broader cognitive delay. While many neurodevelopmental conditions are not diagnosed until the toddler, preschool, or school age stages, DS is diagnosed prenatally or at birth, which allows for the prospective study of early developmental processes. Identifying early factors related to the onset of social development heterogeneity may in very young children with DS, therefore, may have broader relevance for the study of early social vulnerabilities in the general population of children with intellectual disability.

Moreover, from a translational perspective, identifying early factors that set the stage for more pronounced social challenges in DS can inform anticipatory, tailored interventions (53). If the association between early EF and social foundations is borne out, it may be possible to provide early enrichments that focus on strengthening early inhibitory aspects of attention, early visual engagement and disengagement, and other EF precursor dimensions, which could facilitate the acquisition of both social and cognitive regulatory skills. Such findings could contribute to the rationale for timely early EF intervention for young children with DS, with potential implications beyond established outcomes, like kindergarten readiness and academic achievement (54). Recent work has aimed to remove barriers to participation in EF interventions for children with DS by tailoring interventions to account for the landscape of strengths and challenges generally associated with DS (55). The present study provides an additional rationale for taking such an anticipatory and tailored approach to better meet the needs of young children in this population.

### Limitations

Despite the novel contributions of this work, the present study has several limitations that must be taken into account when interpreting study findings. First, although the emphasis of this study is on understanding potential sources of within-DS variability in social development, a whole-group study design was implemented to evaluate associations with EF ratings. Additional investigation of within-DS heterogeneity through the use of mixture modeling could shed further light on the extent to which individual differences can be understood as representing distinct profiles cross-sectionally and longitudinally. This inquiry is beyond the scope of the present study design, but would constitute a potentially informative next step in the study of these critical early constructs.

Another key factor to consider in the interpretation of study findings is the caregiver-reported nature of the measures analyzed. The EF and social communication assessments were proxy-report measures, which have both advantages and disadvantages. The benefits of proxy-report measures are the potential for a more ecologically valid and comprehensive account of skills and abilities beyond what can be observed in a direct assessment setting for a brief period of time. However, the inherently subjective nature of caregiver ratings also has limitations in that different caregivers may interpret and understand their children in unique ways from one another, which cannot be controlled for within analytic models. Previous work has, in fact, reported at least moderate associations between caregiver ratings and direct EF assessment in school age children with DS (20). Future research should expand on this topic and evaluate the link between regulatory vulnerabilities and social communication outcomes in DS via direct laboratory-based measurement. It is also noted that the Regulation subscale was not related to social foundations in the present sample. It may be the case that the presence or absence of more intense emotional displays has no bearing on the acquisition of early social foundations, but it is also noted that the Regulation scale had the highest average item score, and these null results could be attributed to a restricted range.

Some consideration of the item level ratings for the CSBS is warranted as well. Of the 24 CSBS items, five demonstrated negative and two items demonstrated positive skew. This information is clinically and developmentally informative for characterizing early presentations in DS but could raise concerns regarding composite total range. Skewed items in certain areas would be expected for developmentally sequenced skills in toddler samples, wherein some behaviors are nearly universal and others are only beginning to emerge. Variability in composite scores was sufficient for subsequent analyses, suggesting that even with a subset of skewed items, the CSBS is captures a meaningful range of performances in toddlers with DS at this CA range.

Another important limitation of the present study involves the developmental chronological window of participants in the study. Ideally, all participants would have been evaluated at the same precise CA of 18 months. However, given the relatively low incidence of DS, and the logistical barriers often faced by families to find time and resources to participate in research studies, accommodations were made to include participants within a somewhat wider CA window. As such, participants who were 17 months were included in a sample with participants up to 24 months of age. Although this time window is quite narrow relative to most other DS research participant samples, some degree of heterogeneity in the participants observed may have been attributed to CA, rather than the key dimensions of interest. It may also be important to consider the range of developmental ages observed in this sample. Though the majority of participants had MAs near 12 months, estimates ranged between 4 months and 20 months. Notably, Bayley cognitive status was controlled for in the regression models, as a way to evaluate the association between EF and social foundations over and above their association with overall developmental status.

Recruitment efforts for the present study attempted to reach a representative sample of toddlers with DS across a variety of demographic dimensions. However, some response bias may be present in the sample, given that many families were recruited through advocacy groups or targeted recruitment via social media. Participation in community organizations and access to technology likely varies within the population of families of young children with DS, and families who are navigating complex biomedical conditions for their child certainly face more barriers to research participation due to more intense caregiving demands. Therefore, despite efforts to obtain a large and representative sample of infants with DS, the present study likely overrepresented families with access to resources and who receive supports through social and organizational entities as well as children with less biomedical complexity. Similarly, the sample included in the present study was predominantly white and not Hispanic/Latino. Though the demographics of the sample are reflective of the areas surrounding the three research sites, it is important to note that the findings reported may not be representative of the broader population.

### Conclusion

Overall, findings from this study suggest that individual differences in social adaptation may be linked to early developing EF skills in young children with DS. Difficulties in acquiring social communication and play skills could have cascading effects on developing social competencies and adaptive functioning more broadly. These findings may inform future direct assessment-based study designs that can further evaluate the relationship between these emerging skills in carefully controlled laboratory settings. Ultimately, characterizing the wide range of social development outcomes and their potential underpinnings advances our understanding of phenotypic emergence in DS, and potentially other neurogenetic conditions associated with intellectual disability. This focus on characterizing the full range of outcomes in DS may also inform the refinement of more tailored approaches to early interventions that can promote stronger foundations from the earliest developmental stages.

## Data Availability

The raw data supporting the conclusions of this article will be made available by the authors, without undue reservation.
