# Supplementary material for: Caregiver-reported social foundations and cognitive regulation in toddlers with Down syndrome
Source: Front Psychiatry. 2026 Jul 1;17:1803350. doi: 10.3389/fpsyt.2026.1803350 (PMC13370935; doi:10.3389/fpsyt.2026.1803350)
Supplement: Supplementary file 1 [file SupplementaryFile1.docx]

**Supplemental Table 1**

*CSBS Item Summary*

| CSBS Item | | Mean | SD | Skew |
| --- | --- | --- | --- | --- |
| 1 | Caregiver knows when child is happy and when child is upset | 1.98 | 0.14 | -7.01 |
| 2 | Child looks at caregiver while playing with toys | 1.55 | 0.54 | -0.57 |
| 3 | Child smiles or laughs when looking at caregiver | 1.94 | 0.23 | -3.81 |
| 4 | Child looks when caregiver points to a toy across the room | 1.23 | 0.56 | 0.02 |
| 5 | Child lets caregiver know that he/she needs help or wants an object out of reach | 1.38 | 0.70 | -0.67 |
| 6 | Child tries to get caregiver’s attention | 1.60 | 0.56 | -1.01 |
| 7 | Child does things just to get caregiver to laugh | 1.06 | 0.71 | -0.08 |
| 8 | Child tries to get caregiver to notice interesting objects | 0.78 | 0.74 | 0.38 |
| 9 | Child picks up objects and gives them to caregiver | 1.37 | 0.73 | -0.70 |
| 10 | Child shows objects to caregiver without giving the object | 1.17 | 0.72 | -0.26 |
| 11 | Child waves to greet people | 1.45 | 0.66 | -0.78 |
| 12 | Child points to objects | 0.93 | 0.78 | 0.11 |
| 13 | Child nods his/her head to indicate yes | 0.34 | 0.63 | 1.64 |
| 14 | Child uses sounds or words to get attention or help | 1.58 | 0.57 | -0.93 |
| 15 | Child string sounds together | 1.19 | 0.81 | -0.35 |
| 16 | Child makes consonant sounds | 2.35 | 0.87 | -0.38 |
| 17 | Child uses multiple different words meaningfully | 1.10 | 0.90 | 0.57 |
| 18 | Child puts two words together | 0.12 | 0.36 | 2.89 |
| 19 | Child responds when name is called | 1.69 | 0.50 | -1.25 |
| 20 | Child understands different words or phrases | 1.72 | 1.05 | 0.33 |
| 21 | Child shows interest in playing with a variety of objects | 1.92 | 0.28 | -2.95 |
| 22 | Child uses multiple different objects appropriately | 2.35 | 1.06 | -0.24 |
| 23 | Child stacks multiple blocks or rings | 1.12 | 1.05 | 0.43 |
| 24 | Child engages in pretend play | 0.50 | 0.68 | 0.97 |

*Note*. Table includes paraphrased CSBS items.

**Supplemental Table 2**

*EEFQ Item Summary*

| EEFQ Item | | Mean | SD | Skew |
| --- | --- | --- | --- | --- |
| IC 1 | Stops reaching for something entirely after hearing "no/don't touch" | 3.06 | 1.33 | 0.48 |
| IC 2 | Pauses for a second after hearing "no/don't touch” | 4.12 | 1.69 | -0.11 |
| IC 3 (r) | Reaches for things they've repeatedly been told not to touch (e.g., outlets, oven) | 3.50 | 1.62 | 0.26 |
| IC 4 | Slows down and tries an action carefully (e.g., fitting a shape sorter piece, catching a dangling toy) | 4.17 | 1.67 | -0.21 |
| IC 5 | Repeats a new skill until they master it (e.g., grabbing a toy that is out of reach) | 5.05 | 1.24 | -0.35 |
| IC 6 | Sticks with a tricky task for a long time | 4.03 | 1.51 | -0.20 |
| IC 7 | Quiets down when "shushed" in public settings | 3.59 | 1.54 | -0.03 |
| Flex 1 | Explores most or all features of a multi-part toy | 4.69 | 1.36 | -0.40 |
| Flex 2 (r) | Plays the same repetitive way with a toy, without varying their approach (e.g., only spinning car wheels) | 4.33 | 1.44 | -0.01 |
| Flex 3 | Tries a different approach to a tricky task on their own (e.g., testing other holes in a shape sorter) | 3.41 | 1.60 | 0.27 |
| Flex 4 (r) | Keeps repeating the same unsuccessful action even after being shown a different one | 4.30 | 1.31 | -0.02 |
| Flex 5 | Uses everyday objects to solve problems unprompted (e.g., dragging over a box to climb) | 2.8 | 1.60 | 0.62 |
| Flex 6 | Identifies the odd one out in a group of items | 2.26 | 1.56 | 1.10 |
| Flex 7 | Stares at, points to, or touches something new for an extended time | 3.73 | 1.67 | -0.07 |
| WM 1 | Follows a simple instruction for a task they're interested in, without getting distracted | 3.74 | 1.66 | -0.21 |
| WM 2 (r) | Seems to forget what they were doing partway through | 5.60 | 1.15 | -0.43 |
| WM 3 | Resumes an activity after a minor interruption | 4.65 | 1.27 | -0.49 |
| WM 4 | Repeats or copies something just demonstrated to them | 4.42 | 1.31 | -0.13 |
| WM 5 | Notices when something given to them earlier is missing or changed | 3.89 | 1.49 | -0.09 |
| WM 6 | Pursues something they want even after watching it get hidden | 5.88 | 1.14 | -1.36 |
| Reg 1 | Calms within 3 minutes after a minor frustration | 6.17 | 1.03 | -1.86 |
| Reg 2 (r) | Stays upset (crying, withdrawn) for over a minute after being denied something | 5.17 | 1.40 | -1.02 |
| Reg 3 (r) | Stays upset (crying, withdrawn) for over a minute after being pulled away from something fun | 5.49 | 1.41 | -1.12 |
| Reg 4 (r) | Is upset (crying/whimpering, withdrawn) when unable to do something | 5.09 | 1.22 | -0.48 |
| Reg 5 (r) | Is angry (screaming, banging) when unable to do something | 5.56 | 1.39 | -0.84 |
| Reg 6 (r) | Stays angry (screaming, shouting, lashing out) for over a minute after being denied something | 6.13 | 1.10 | -1.39 |
| Reg 7 (r) | Stays angry for over a minute after being pulled away from something fun | 6.10 | 1.26 | -1.84 |
| Reg 8 (r) | Becomes upset during a fun activity and needs soothing. | 6.14 | 0.79 | -0.59 |

*Note.* Table includes paraphrased EEFQ items. (r) indicates a reverse-scored item; IC = Inhibitory Control; Flex = Flexibility; WM = Working Memory; Reg = Regulation.

**Supplemental Table 3**

*Correlations Among EEFQ Subscales and CSBS Scores With 95% Confidence Intervals*

| Variable | 1 | 2 | 3 | 4 | 5 | 6 | 7 | 8 | 9 | 10 | 11 | 12 | 13 |
| --- | --- | --- | --- | --- | --- | --- | --- | --- | --- | --- | --- | --- | --- |
| 1. EEFQ IC |  |  |  |  |  |  |  |  |  |  |  |  |  |
| 2. EEFQ Flex | 0.66*** [.54, .76] |  |  |  |  |  |  |  |  |  |  |  |  |
| 3. EEFQ WM | 0.46*** [.30, .60] | 0.53*** [.38, .66] |  |  |  |  |  |  |  |  |  |  |  |
| 4. EEFQ Reg | 0.15 [-.04, .33] | 0.17 [-.02, .35] | 0.06 [-.13, .25] |  |  |  |  |  |  |  |  |  |  |
| 5. CSBS Emo | 0.26* [.07, .43] | 0.33*** [.15, .49] | 0.16 [-.03, .34] | -0.03 [-.22, .16] |  |  |  |  |  |  |  |  |  |
| 6. CSBS Comm | 0.46*** [.29, .60] | 0.45*** [.28, .59] | 0.3** [.11, .46] | 0.05 [-.15, .23] | 0.48*** [.32, .61] |  |  |  |  |  |  |  |  |
| 7. CSBS Gest | 0.43*** [.26, .57] | 0.46*** [.29, .59] | 0.28** [.10, .45] | 0.02 [-.17, .21] | 0.55*** [.41, .67] | 0.68*** [.56, .77] |  |  |  |  |  |  |  |
| 8. CSBS Sounds | 0.39*** [.22, .54] | 0.36*** [.18, .51] | 0.21* [.02, .38] | 0.15 [-.04, .33] | 0.29** [.11, .46] | 0.49*** [.33, .62] | 0.34*** [.16, .50] |  |  |  |  |  |  |
| 9. CSBS Use of Words | 0.25* [.07, .42] | 0.25* [.06, .42] | 0.11 [-.08, .30] | -0.17 [-.35, .02] | 0.33*** [.15, .49] | 0.33*** [.15, .49] | 0.43*** [.26, .57] | 0.42*** [.25, .56] |  |  |  |  |  |
| 10. CSBS Und of Words | 0.38*** [.20, .53] | 0.28** [.10, .45] | 0.32** [.14, .48] | 0.03 [-.16, .22] | 0.47*** [.31, .61] | 0.39*** [.21, .54] | 0.49*** [.33, .62] | 0.3** [.12, .46] | 0.42*** [.25, .57] |  |  |  |  |
| 11. CSBS Objects | 0.36*** [.18, .51] | 0.28** [.09, .45] | 0.38*** [.20, .53] | -0.06 [-.24, .14] | 0.32** [.14, .48] | 0.4*** [.23, .55] | 0.58*** [.44, .69] | 0.24* [.06, .41] | 0.35*** [.18, .51] | 0.42*** [.25, .56] |  |  |  |
| 12. CSBS Social | 0.47*** [.31, .61] | 0.5*** [.34, .63] | 0.31** [.12, .47] | 0.02 [-.17, .21] | 0.69*** [.58, .78] | 0.88*** [.83, .92] | 0.93*** [.89, .95] | 0.45*** [.28, .59] | 0.43*** [.26, .57] | 0.51*** [.36, .64] | 0.54*** [.39, .66] |  |  |
| 13. CSBS Speech | 0.4*** [.22, .55] | 0.37***  [.20, .52] | 0.2* [.01, .37] | 0.02 [-.17, .21] | 0.36*** [.18, .52] | 0.5*** [.34, .63] | 0.44*** [.28, .58] | 0.9*** [.86, .93] | 0.77*** [.68, .84] | 0.41*** [.24, .56] | 0.34*** [.16, .50] | 0.52*** [.37, .65] |  |
| 14. CSBS Symbolic | 0.43*** [.26, .57] | 0.33*** [.15, .49] | 0.42*** [.25, .56] | -0.03 [-.22, .16] | 0.44*** [.27, .58] | 0.46*** [.30, .60] | 0.64*** [.51, .74] | 0.31** [.13, .47] | 0.44*** [.28, .59] | 0.74*** [.64, .81] | 0.92*** [.88, .94] | 0.62*** [.49, .73] | 0.43*** [.26, .57] |

*Note.* Significance levels based on FDR-adjusted *p*-values **p* < .05, ***p* < .01, ****p* < .001. IC = Inhibitory Control; Flex = Flexibility; WM = Working Memory; Reg = Regulation; Emo = Emotion and Eye Gaze; Comm = Use of Communication; Gest = Use of Gestures; Sounds = Use of Sounds; Und of Words = Understanding of Words; Objects = Use of Objects.
